# mHealth system (ATOPE+) to support exercise prescription in breast cancer survivors: a reliability and validity, cross-sectional observational study (ATOPE study)

**DOI:** 10.1038/s41598-022-18706-7

**Published:** 2022-09-08

**Authors:** Paula Postigo-Martin, Rocío Gil-Gutiérrez, Salvador Moreno-Gutiérrez, Maria Lopez-Garzon, Ángela González-Santos, Manuel Arroyo-Morales, Irene Cantarero-Villanueva

**Affiliations:** 1 Biomedical Group (BIO277), University of Granada, Granada, Spain; 2 Department of Physiotherapy, Faculty of Health Sciences, University of Granada, Granada, Spain; 3 A02-Cuídate, Instituto de Investigación Biosanitaria ibs.GRANADA, Granada, Spain; 4 Department of Nursing, Faculty of Health Sciences, University of Granada, Avenida de la Ilustración, 60, 18016 Granada, Spain; 5 MP07-Bases Fisiopatología y Terapéutica Médica, Instituto de Investigación Biosanitaria ibs.GRANADA, Granada, Spain; 6 Department of Computer Architecture and Technology, CITIC-UGR, Research Center, University of Granada, Granada, Spain; 7 Unit of Excellence on Exercise and Health (UCEES), University of Granada, Granada, Spain; 8 Sport and Health Research Center (IMUDs), Granada, Spain

**Keywords:** Breast cancer, Fatigue, Quality of life, Diagnostic markers, Predictive markers, Rehabilitation

## Abstract

Physical exercise is known to be beneficial for breast cancer survivors (BCS). However, avoiding nonfunctional overreaching is crucial in this population, as they are in physiological dysregulation. These factors could decrease their exercise capacity or facilitate nonfunctional overreaching, which can increase their risk of additional morbidities and even all-cause mortality. The focus of this study is to evaluate the reliability and validity of the ATOPE+ mHealth system to estimate autonomic balance and specific wellness parameters associated with BCS’ perceived load, thereby informing nonlinear prescriptions in individualized physical exercise programs for BCS.Twenty-two BCS were included in the reliability and validity analysis. Measures were taken for four days, including morning autonomic balance by heart rate variability, self-reported perception of recovery from exercise, sleep satisfaction, emotional distress and fatigue after exertion. Measures were taken utilizing the ATOPE+ mHealth system application. The results of these measures were compared with criterion instruments to assess validity.The reliability results indicated that the intraclass correlation coefficient (ICC) showed an excellent correlation for recovery (0.93; 95% CI 0.85–0.96) and distress (0.94, 95% CI 0.89–0.97) as well as good correlation for the natural logarithm of the mean square root differences of the standard deviation (LnRMSSD) (0.87; 95% CI 0.74–0.94). Sleep satisfaction also showed an excellent correlation with a weighted kappa of 0.83. The validity results showed no significant differences, except for fatigue. ATOPE+ is reliable and valid for remotely assessing autonomic balance, perception of recovery, sleep satisfaction and emotional distress in BCS; however, it is not for fatigue. This highlights that ATOPE+ could be an easy and efficient system used to assess readiness in BCS, and could help to improve their health by supporting the prescription of optimal and safe physical exercise. *Trial registration* NCT03787966 ClinicalTrials.gov, December 2019 [ATOPE project]. https://clinicaltrials.gov/ct2/show/NCT03787966.

## Introduction

### Background

Physical exercise is already known to mitigate the side effects of cancer and its treatment^1^, as well as reduce cancer recurrence^2,3^, and mortality^2^ in breast cancer survivors (BCS). In general, physical exercise should aim to achieve the desired benefits while balancing the risks of suboptimal loading or overtraining. Avoiding nonfunctional overreaching or insufficient recovery would be important for BCS, as they are in a situation of physiological vulnerability due to cancer and its treatment. Their physiological systems have undergone changes due to treatment, such as increased oxidative stress^4^, chronic inflammation^5^, and reduced immune function^6^; which are similar to the alterations present in overtraining in athletes^7^. While these alterations are related to treatment side effects, they may also predispose these women to physiological dysregulation which maintained over time, would decrease their physical exercise assimilation capacity or even lead them to overreaching^8^, and increasing their vulnerability to illness and death^9^.

In oncology, the conventional prescription is linear, with a progressive and standard increase in intensity, frequency and duration parameters^10^. However, a nonlinear approach maximises the adaptation to exercise, which has been suggested to fit best to an optimal and safe dose-recovery period^10^, thus, could be safest for a heterogeneous population such as BCS. Additionally, the presence of nonresponders^11^, a wide range of adherence^12^, and patients with comorbidities and higher toxicities^13^ should be considered in physical exercise programs, which may challenge current physical exercise prescription approaches.

For this matter, nonlinear prescription is usually guided with methods such as heart rate variability (HRV), which allows a better dose adjustment and prevents overtraining^14^. Nevertheless, this has been commonly used in athletes, but its use is not as common in the clinical population (ClinicalTrials.gov Identifier: NCT03745742), and specifically in the oncological population, where prescription is mostly informed by survivors’ symptoms^15^. Therefore, it is of great interest to develop a support tool such as ATOPE+^16^ to assist with a nonlinear prescription, monitor readiness, and control the loading-recovery cycle to allow safe and effective doses following physiological adaptations.

### ATOPE+ mHealth system

When working with a vulnerable population such as BCS, it is important to rely on validated tools. For instance, a previous example would be the BENECA application in BCS^17^, which was successful in terms of reliability^17^ and efficacy^18^. BENECA records energy expenditure based on exercise and food ingested and recommends increasing or decreasing physical activity to maintain energy balance. However, ATOPE+ adds further information in prescribing physical exercise by including physiological readiness information in optimizing exercise dose. ATOPE+ is based on assessing autonomic balance with HRV, as it reflects fatigue, stress and other factors that influence exercise assimilation^7^. However, it has been stated that other internal load parameters are part of novel risks or preclinical alterations preceding overtraining, such as poor sleep, worsened mood, stress, and increased fatigue^7^. These are especially important in patients with cancer and could mediate HRV on their own; therefore, they are also included in ATOPE+.

The gold standard for autonomic balance is the assessment of HRV with an electrocardiogram (ECG). However, for recovery and fatigue, there is a wide range of blood parameters, such as blood lactate concentration^19^ and creatine kinase (CK)^20^; for sleep analysis, it is the use of polysomnography; for stress, cortisol analysis^21^. However, these are not easily accessible and expensive, and some of them are invasive and time-consuming tests. For these reasons, we selected other instruments validated in previous studies as comparisons to validate ATOPE+, including a Holter monitor^22^, Perceived Recovery Status Scale^23^, Sleep Diary^24^, Emotional Distress Thermometer^25^, and Borg CR-10 Scale^26^. ATOPE+ HRV information, complemented with other self-reported parameters, can remotely engage oncological populations. Therefore, ATOPE+ is HRV-guided as well as complemented with other internal load parameters to remotely monitor the oncological population.

### Aim

The aim of this study was to evaluate the reliability and validity of the ATOPE+ application to estimate autonomic balance by HRV and wellness parameters to inform nonlinear individualized physical exercise prescription for posttreatment BCS.

## Methods

A cross-sectional observational study was conducted to test the reliability and validity of ATOPE+ with 22 BCS.

### Participants

Potential participants were identified from the referrals received from the Surgical Unit of the Hospital Universitario Clínico San Cecilio in Granada, Spain, between February and August 2021. BCS were eligible if they had been diagnosed with breast cancer (stages I-III), had to have basic mobile phone capabilities, and had at least one year since the end of oncological treatment (hormonal treatment was not an exclusion criterion). In contrast, potential participants were excluded if they had not finished chemotherapy or radiotherapy at least one year before the study start date, had psychiatric or cognitive disorders that prevented from following the instructions of the protocol given, or did not have access to a smartphone.

Eligible women were asked to come to the CUIDATE group’s facilities. A member of the research group explained the assessment protocol and installed ATOPE+ on their mobile phones. They were asked to use ATOPE+ in the presence of a researcher to ensure correct assessment performance. They were also given the materials needed for remote assessment (i.e., ECG device, chest strap, questionnaires and assessments instructions).

### Sample size

A sample size of 20 participants was estimated to be necessary to identify an intraclass correlation coefficient (ICC) of 0.8 between the mean square root differences of the standard deviation (LnRMSSD) assessed with the Polar H10 chest band and the ECG (Gold Standard), 90% power, and an alpha error of 0.5^27^. Considering a potential 10% dropout rate, 22 BCS were recruited for the study.

### Description of ATOPE+ and data collection

**Figure 1:**
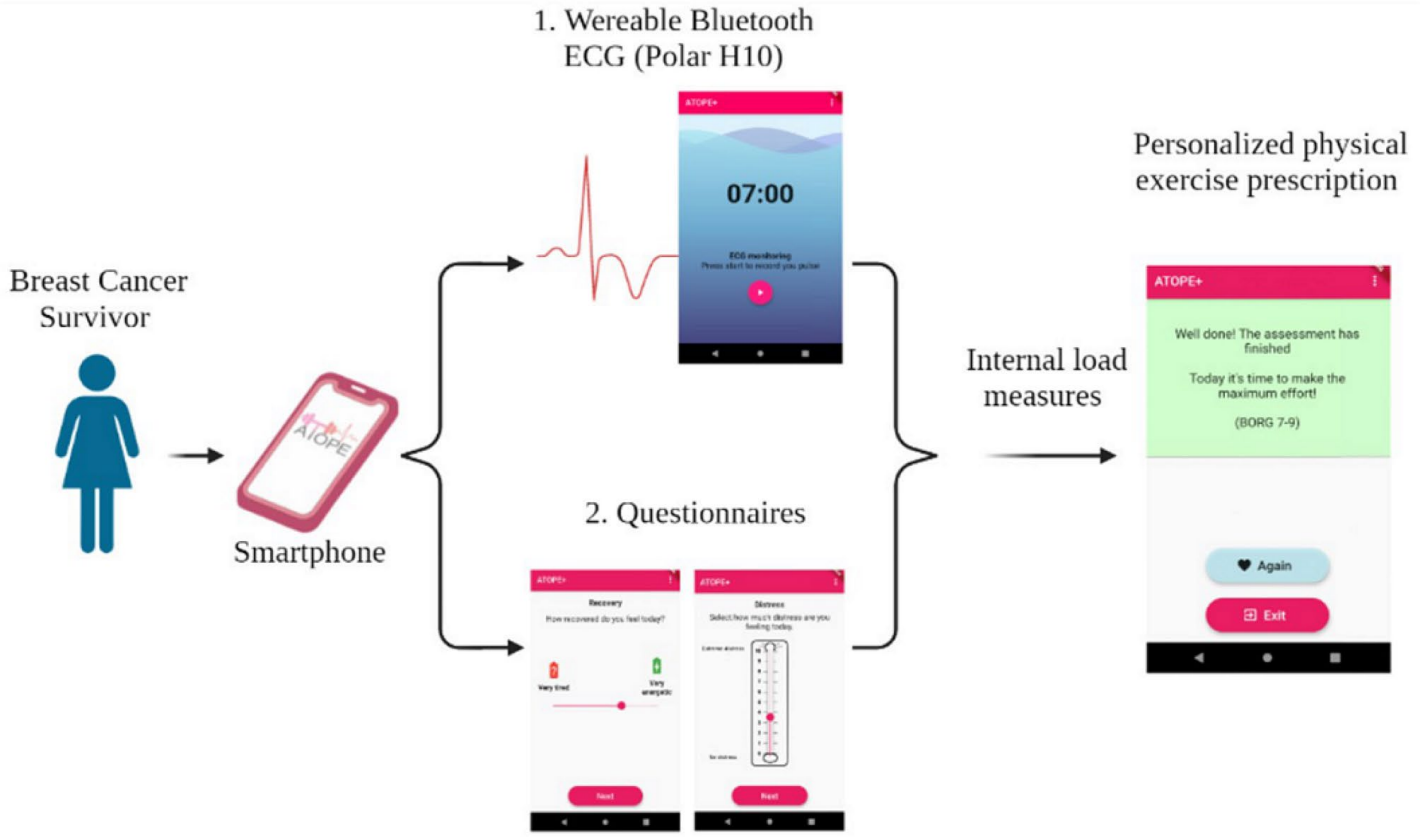
ATOPE+ mHealth system overview. Created with Biorender.com.

To complete the study, patients had to take measurements with ATOPE+ and their comparison instruments (Table 1): Holter monitor^22^, Perceived Recovery Status Scale^23^, Sleep Diary^24^, Emotional Distress Thermometer^25^, and Borg CR-10 Scale^26^, during four consecutive mornings, including one weekend day in order to be as precise to normal routine as possible. Patients were told to follow a normal sleep routine during the study. Once they finished the application protocol, they continued filling out the comparison questionnaires given in paper format and the sleep diary. An overview of the ATOPE+ mHealth system is shown in Fig. 1.

**Table 1:** ATOPE+ and criterion instruments details.

Outcome	Instruments	Presentation/question
**Autonomic balance**		
Criterion instrument	Holter monitor^22^	Countdown timer
ATOPE+	Polar H10 chest band	
**Perception of recovery**		
Criterion instrument	Perception of Recovery Scale^23^ Horizontal 100-mm numerical scale Seven descriptors from very tired to very energetic	Participants were presented the scale and are asked to estimate their perceived level of recovery
ATOPE+	Adapted from the criterion instrument: Horizontal visual analogue scale Two descriptors at both end (Very tired-very energetic)	“How recovered do you feel today?”
**Sleep satisfaction**		
Criterion instrument	Subscale of quality of sleep from the sleep diary^24^ Five options: very bad, bad, normal, good, very good	“How was your quality of sleep last night?”
ATOPE+	Adapted from the criterion instrument Horizontal visual analogue scale Two descriptors at both ends (Not at all satisfied-Very satisfied)	
**Emotional distress**		
Criterion instrument	NCCN emotional distress thermometer^25^ Vertical numerical scale Two descriptors at both ends (no distress, extreme distress)	Participants were asked to circle the number that best describes the emotional distress that they experience
ATOPE+	Adapted from the criterion instrument Vertical numerical scale Two descriptors at both ends (no distress, extreme distress)	“Select how much emotional distress are you feeling today”
**Fatigue**		
Criterion instrument	Borg CR-10 scale^26^ Eight descriptors from no fatigue to extreme fatigue	“How much fatigue do you feel after the Sit to Stand test?”
ATOPE+	Adapted from the criterion instrument: Horizontal visual analogue scale Two descriptors at both ends (no fatigue, extreme fatigue)	

*NCCN* National Comprehensive Cancer Network; *LNRMSSD* mean square root differences of the standard deviation; *STS* Sit to Stand Test.

ATOPE+ was developed by the Biomedical (BIO-277) ‘CUIDATE’ research group and the Department of Computer Architecture and Technology, CITIC-UGR Research Centre, both from the University of Granada, Spain. The development of ATOPE+ is part of the ATOPE project^28^, registration number NCT03787966 ClinicalTrials.gov, December 2019.

The ATOPE+ mHealth system is composed of a cross-platform application (Android/iOS) and a centralized secure server. The application provides patients with an interface to record their HRV and to report their wellness through questionnaires. The centralized secure server enables data storage and processing, as well as the generation of tailored exercise prescription according to expert rules. The architecture and usability of ATOPE+ have previously been described^16^. The registration code of the system is 1710092555522.

Once the research team has installed the application on the participant’s phones and created their personal profiles, patients were ready to start using the application. In the main view, patients were able to read a quick tutorial of how to perform the assessment or start it. The measurement started once they pushed the “Start” button, so they had to be prepared before pushing the button. The assessment of the HRV was first. A notification with sound and vibration alerted the participant that this first step was completed, and the rest of the protocol continued.

Perceived recovery, sleep satisfaction and fatigue were assessed with horizontal continuous Likert scales from 0 to 10 with labels in the values at the extremes and a continuous slider included in ATOPE+. For emotional distress, the scale was positioned vertically. The final part of the assessment consisted of performing 10 repetitions of the "Sit To Stand Test" (STS) and assessing the fatigue perceived after the effort with a rating of perceived exertion scale from 0 to 10. After that, the evaluation was completed. The answers were sent to the server, and the participant received an automatic personalized message about their readiness for either a high-intensity session, a moderate-intensity session, or active recovery. More information about the intervention was published on a previous protocol^28^.

### Comparison instruments

#### Autonomic balance

Autonomic balance was assessed with ATOPE+ and a Polar H10 chest strap (Polar H10, Polar Electro Oy, Kempele, Finland) connected through Bluetooth and was compared with an ECG (Norav Holter DL800, Braemar Inc, Eagan, EEUU) monitor, which is considered the gold standard. From a 7-min recording, the first and last minutes were cut off to achieve clear and precise interpretations of vagal tone with a 5-min signal, as recommended by the Task Force of the European Society of Cardiology and the North American Society for Pacing and Electrophysiology^29^. The time domain parameter rMSSD (the square root of the mean squared differences) was analysed.

For ATOPE+ , data were exported to a computer for analysis. As recommended by the Taskforce, all artifacts (ectopic beats, arrhythmic events, and noise effects) in the RR time series were corrected or removed to reduce the chances of substantial deformities that can occur in HRV analysis^30^. In the case of Holter monitor data, NH300 software (Norav, version 3.0, 2009, Norav Medical Ltd) was used to perform the spectral analysis by using Fast Fourier transform algorithms to remove noise from recordings. The sampling rate was 128 samples/second. The frequency filter was set from 0.05 to 60 Hz. Due to low sampling rate, the software itself applied an interpolation algorithm to improve R peak detection^31^.

After waking up and emptying their bladder, participants were instructed to moisten and place the chest band and the ECG monitor. Then, lying on their beds facing the ceiling, data recording was performed under the same terms of duration for both devices.

#### Perception of recovery

The Perception of Recovery Scale was used as a comparison to assess the perception of recovery. It is a subjective self-administered Likert-type scale with scores from 1 to 10 (Table 1) and with a sensitivity and specificity of 0.82 and 0.81, respectively^23^.

#### Sleep satisfaction

As a comparison instrument, the subsection of quality of sleep from the consensus sleep diary, a reliable tool for prospectively measuring quality of sleep^24^, was used. It is a self-reported method that includes quantitative and qualitative aspects related to each night of rest (Table 1). This method, compared to polysomnography, has a kappa coefficient of 0.87^32^.

#### Emotional distress

The Emotional Distress thermometer according to “The NCCN Clinical Practice Guidelines in Oncology” was used as a comparison to measure emotional distress. This tool consists of a Likert-type scale with values from 0 to 10, where 0 is "no emotional distress" and 10 constitutes "extreme emotional distress" (Table 1). In the Spanish oncology population, this thermometer has a sensitivity of 0.9 and a specificity of 0.64^33^.

#### Peripheral fatigue

The Borg-CR 10 scale was used as comparison for the evaluation of the perceived level of fatigue after physical exertion. After performing 10 repetitions at a rhythm of 40 beats per minute (marked by a metronome included in ATOPE+) of the STS, a test frequently used as a protocol to induce fatigue in the lower extremities, participants completed this questionnaire, which consists of scores from 0 to 10 ("Not at all" to "Very, very hard", respectively) (Table 1). This scale has a reliability of 0.66 according to the kappa coefficient in the clinical population of women^26^.

### Statistical analysis

A descriptive analysis was performed to summarize sociodemographic and clinical characteristics of participants. Continuous variables are expressed as the mean ± standard deviation, and categorical variables are expressed as numbers and percentages. The normal distribution of the variables was checked by means of the Shapiro-Wilks test. Data that did not follow a normal distribution were transformed into Ln(x) or Ln(x + 1) to enable parametric analysis. All analyses were carried out by a blinded researcher.

IBM SPSS version 24 was used for all analyses (IBM Statistical Program for Social Sciences SPSS Statistic, Corp., Armonk, New York). Bland–Altman analyses were carried out in order to properly establish  agreement^34^ between ATOPE+ methods and Gold Standard methods by using Excel worksheets (Microsoft Excel version 16.55, Microsoft, Washington, EEUU). A 95% Confidence Interval (CI) was established, and significance was set at *p* < 0.05.

### Reliability

For each outcome measure, concordance between comparison instruments and those included in ATOPE+ was calculated. Bearing in mind that Pearson correlation coefficients, paired t test, and Bland–Altman plots are methods for analysing agreement but not ideal in terms of reliability^35^, interdevice ICC were calculated to reflect relative reliability (Table 2). ICC scores were categorized as poor (< 0.5), moderate (0.5–0.75), good (0.75–0.90) and excellent (> 0.90)^36^. Weighted kappa was used for categorical variables. The suggested interpretation for agreement is as follows: ≤ 0 poor, 0.01–0.20 slight, 0.21–0.40 fair, 0.41–0.60 moderate, 0.61–0.80 substantial, and 0.81–1 almost perfect^37^. Additionally, the standard error of measurement was calculated. These calculations identified within subject variation for each method, indicating the magnitude to which repeated measures changed for participants.

**Table 2:** Indices of reliability of ATOPE+mHealth system for mean HRV parameter, recovery, sleep, emotional distress and fatigue of breast cancer survivors (N = 21).

Outcome	SEm; mean value (lower and upper estimated true score)	Mean ICC (95% CI)
Autonomic balance (LnRMSSD)	0.11; 3.79 (3.47, 4.11)	0.87 (0.74 to 0.94)
Perception of recovery (points)	0.43; 5.93 (5.09, 6.77)	0.93 (0.85 to 0.96)
Sleep satisfaction (points)	–	0.83^a^
Emotional distress (points)	0.40; 2.75 (1.97, 3.52)	0.94 (0.89 to 0.97)
Fatigue (points)	0.61; 3.67 (2.48, 4.85)	0.86 (0.29 to 0.95)

*ICC* intraclass correlation coefficient (95% confidence interval); *LnRMSSD* natural logarithm of the mean square root differences of the standard deviation; *SEm* standard error of measurement.

^a^Weighted kappa.

### Validity

To determine the validity of ATOPE+, paired samples t tests were conducted comparing ATOPE+ measurements versus reliable measurements. Continuous variables were analyzed by Student's t-test in the case of normal parametric variables, and nonparametric variables were analyzed with Wilcoxon test. Considering that they only reflect proportional relationships and can cause erroneous interpretation of measurements, to establish the agreement between the comparison instruments and ATOPE+ methods, Bland–Altman analyses were also carried out, which allowed us to see the difference between two clinical measurement devices against each method’s mean. To obtain further information, sleep satisfaction was treated as a continuous variable for this purpose. To establish interdevice agreement, Cohen's d for effect size was used, with effect sizes categorised as follows: 0 to 0.19, trivial; 0.2 to 0.59, small; 0.6 to 1.19, moderate; 1.2 to 1.99, large; and > 2.0, very large^38^. The Wilcoxon rank test and effect size were calculated for ordinal variables.

### Ethics approval and consent to participate

This study was approved by the ‘Ethics Committee of Biomedical Research of Granada’ (Granada, Spain) (0507-N-18, July 27, 2018). All participants received written and verbal information. Informed consent was obtained from all participants by signing a specific document for this purpose. All methods were carried out in accordance with the Declaration of Helsinki.

## Results

### Sample description

A total of 22 BCS who had finished oncological treatment at least one year ago were recruited for the study. Of these participants, 1 could not be included in the sample because she was not able to complete the four days of measurement due to personal issues. The mean age of the participants was 49.48 (SD 8.38) years. Tables 3 and 4 summarize demographic and clinical characteristics of the participants. Of the participants, 6 (27.27%) were unemployed. Most participants had stage II breast cancer (36.36%) and had undergone surgery, chemotherapy, and radiotherapy as treatment (63.64%).

**Table 3:** Demographic characteristics (N = 22).

Characteristic	Participants
Age (years), mean (SD)	49.48 (8.38)
**Race, n (%)**	
Caucasic	20 (90.91)
Other	1 (4.55)
Missing	1 (4.55)
**Social situation, n (%)**	
Married	14 (63.64)
Single	4 (18.18)
Divorced	2 (9.10)
Widowed	1 (4.55)
Missing	1 (4.55)
**Occupation, n (%)**	
Currently working	5 (22.73)
Her duties	3 (13.64)
Current sick leave	4 (18.18)
Unemployed	6 (27.27)
Retired	1 (4.55)

*SD* standard deviation.

**Table 4:** Clinical characteristics (N = 22).

Characteristics	Participants
**Menopause, n (%)**	
Premenopause	9 (40.91)
Postmenopause	12 (54.55)
Missing	1 (4.55)
**Medical treatment, n (%)**	
Surgery and chemotherapy	2 (9.10)
Surgery and radiotherapy	3 (13.64)
Surgery, chemotherapy and radiotherapy	14 (63.64)
Missing	1 (4.55)
**Cancer stage, n (%)**	
I	5 (22.73)
II	8 (36.36)
III	4 (18.18)
Missing	5 (22.73)

### Reliability

#### Interclass correlation

The ICC for each comparison instrument and ATOPE+ methods showed evidence of good reliability, with all values higher than 0.86 (Table 2). Sleep satisfaction showed a strong correlation (weighted kappa = 0.87).

### Validity

Validity analysis outcomes are shown in Table 5. The paired sample T-test revealed significant differences for fatigue (*p* < 0.001). The strongest parameter agreement for ATOPE+ compared to comparison instruments was the mean Emotional Distress, with a Pearson correlation of 0.91. In contrast, the weakest parameter agreement with a Pearson correlation of 0.80 was found in LnRMSSD (Table 5).

**Table 5:** Indices of validity for ATOPE+ mHealth system in BCS (N = 21).

Outcome		Value		*p* value	Mean difference between instruments in units of measurement (95% CI)	Pearson/Spearman Correlation (*r*)	Effect size
Instruments	ATOPE+ instrument						
	Comparison instrument						
**Autonomic balance (LnRMSSD)**							
Instruments	ATOPE+ + Polar H10 chest band (mean±SD)	3.79±0.44		0.070	− 0.15 (− 0.60 to 0.26)	0.80	− 0.379 (− 0.818, 0.068)
	Holter^22^ (mean±SD)	3.94±0.42					
**Perception of recovery (points)**							
Instruments	ATOPE+ Likert scale (mean±SD)	5.93±1.62		0.190	0.32 (− 1.39 to 1.88)	0.88	0.283 (− 0.157, 0.716)
	Perfecption of Recovery Scale^23^ (mean±SD)	5.61±1.86					
**Sleep satisfaction (n, %)**							
Instruments	ATOPE+ Likert scale (n, %)	Very bad	1 (4.55)	0.157	–	0.81	− 0.308
		Bad	1 (4.55)				
		Fair	12 (54.55)				
		Good	6 (27.27)				
		Excellent	1 (4.55)				
		Missing	1 (4.55)				
	Sleep diary^24^ (n, %)	Very bad	1 (4.55)				
		Bad	1 (4.55)				
		Fair	14 (63.64)				
		Good	3 (13.64)				
		Excellent	2 (9.091)				
		Missing	1 (4.55)				
**Emotional** **distress** **(points)**							
Instruments	ATOPE+ Likert scale (mean±SD)	2.75±2.4		0.22	0.20 (− 1.22 to 1.62)	0.91	0.244 (− 0.193, 0.676)
	Emotional Distress thermometer of the NCCN^25^ (mean±SD)	2.55±2.39					
**Fatigue (points)**							
	ATOPE+ Borg CR-10 (mean±SD)	3.67±2.22		<.001	1.25 (− 0.80 to 3.31)	0.88	1.323 (0.724, 1.905)
	Borg CR-10 Scale^26^ (mean±SD)	2.41±2.19					

*LnRMSSD* mean square root differences of the standard deviation.

**Figure 2:**
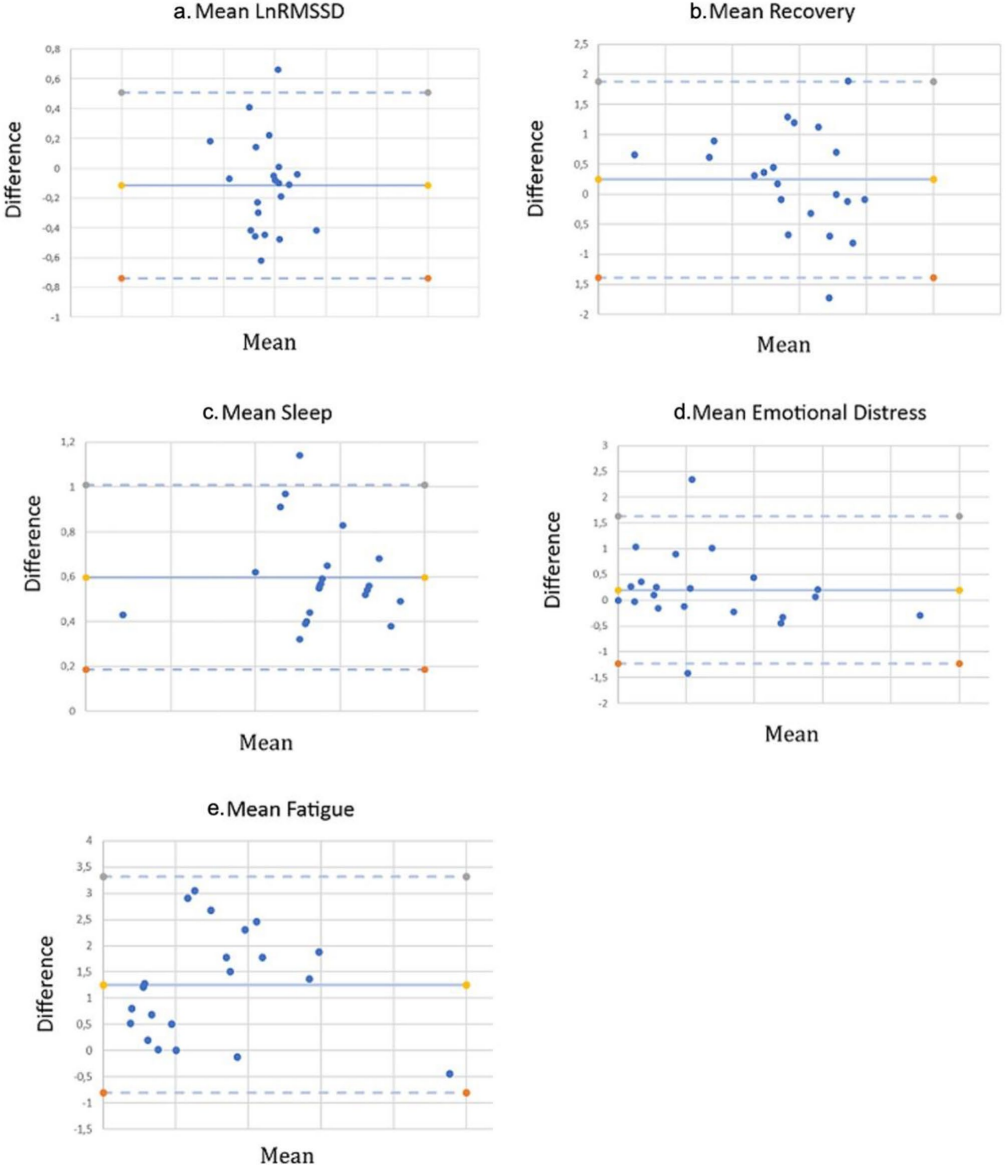
(**a**–**e**) Bland-Altman scatterplots created in order to assess agreement between ATOPE+ methods and Gold Standard methods for HRV parameters, recovery, sleep, emotional distress and fatigue of BCS.

Bland–Altman plots were also generated (Fig. 2a–e), as a graphical representation to depict the difference and limits of agreement between ATOPE+ mean measurement methods and comparison instruments mean measurement methods. Bland-Altman bias, with 95% limits of agreement (LOA), 95% CIs and effect sizes are shown in Table 5. The effect size was small for all variables except for fatigue, which was large.

## Discussion

Our findings showed that ATOPE+ is reliable and valid for assessing autonomic balance, perception of recovery, sleep satisfaction and emotional distress in BCS, with the exception of detecting fatigue. These results highlight that ATOPE+ could be an easy and efficient system to measure tailored readiness in BCS and a tool to improve health by helping professionals to prescribe optimal and safe exercise doses. Moreover, ATOPE+ may provide reliable data-driven analysis with machine learning algorithms, as originally described in its architecture^16^.

### Comparison with prior work

The majority of previous work is not oriented to the clinical population but to athletes^39^ to avoid overtraining^14^ and increase performance^40^. In the clinical population, to our knowledge, a similar tool has not been developed, although there is an ongoing one on post myocardial infarction (ClinicalTrials.gov Identifier: NCT03745742), with less demanding purpose but more oriented to improve functional capacities and reduce fatigue. To our knowledge, there is not an application that has yet been specialized in the oncological population, particularly in women with breast cancer, that has HRV as the principal assessment but complemented with other internal load parameters.

Regarding the validity observed in HRV parameters, the results from ATOPE+ were similar to those in the literature^39,41^. On the one hand, these positive results in ATOPE+ regarding HRV were expected, as the Polar H10 chest band already has had excellent results in the literature measuring this specific outcome^42^. In the study by Gilgen-Ammann et al.^42^, they found that it has excellent validity compared to an ECG monitor and recommended it as gold standard, especially during exercise, as it surpassed the ECG in terms of inducing less recording noise. In addition, good results in our study could have been due to the patients being instructed that it was of great importance to empty their bladder, to remain still during the measurement, to breathe normally, and to have a comfortable environment without distractions. Nevertheless, the correlation was expected to be higher. These results could be obtained because the software that automatically analyses ECG data could not be using the same interpolation methods or selection of outliers or ectopic beats. On the other hand, for the Bland–Altman analysis, previous studies^39,41^ obtained a higher percentage of values of HRV outside the limits of agreement. ATOPE+ reduced percentage of values outside the levels of agreement for HRV, which could have been the result of the application having a timer that told participants where to stop both devices at the same time, as longer samples had been identified to modify HRV indices^29^.

Considering the rest of the parameters, we found significant differences between the fatigue measured with ATOPE+ and the Borg CR-10 Scale, but not for the rest of the internal load parameters. Therefore, it may not be useful for detecting fatigue. Patients were instructed to immediately complete the questionnaires on paper, however, the time in between could explain the differences because as time passes, the perceived fatigue decreases^43^. Another possible hypothesis is that the ATOPE+ fatigue scale may be completed with more verbal anchors, facilitating patients’ answers, or it could be due to differences in the formats used. Therefore, we still wanted to address that even if criteria validity was not met, analysis was performed until the end and found excellent correlation results. In the future, we could add more anchor words or turn the scale horizontal to try to investigate this difference. However, as recovery could be seen as inversely proportional to fatigue, it could be still recognised that having the recovery data may be sufficient from a clinical point of view.

## Limitations and strengths

The system is aimed at BCS and not patients with other types of cancer. Patients had to have basic mobile phone capabilities. In addition, ATOPE+ may be restricted to the available technology and, even if not particularly expensive, could not be accessible for everyone (Polar H10 chest band). The system is only supported in smartphones, not in tablets or computers, and some sight problems in elderly patients could demand family support. In addition, Spanish is the only available language of the system. Additionally, a limitation is that we did not include biomarkers that could support the results, as we wanted a fully noninvasive assessment. In the future, we could establish new tools for different cancer types, have English as an available language, and include photoplethysmography for greater accessibility to the population. To improve individualized physical exercise prescription and to find concordance between subjetctive methods (such as perceived rating of exertion or perceived fatigue, repetitions in reserve) and objective methods (such as heart rate) to control physical exercise intensity, we think it may be interesting the inclusion of invasive biomarkers such as exerkines (specifically, lactate), CK, or maximal oxygen comsumption (VO2max) as an optional complement to ATOPE+ .

ATOPE+ also presents some strengths. The system could be a very powerful tool for professionals, as it may guarantee safe exercise doses. Additionally, it saves time, as readiness or recovery could be assessed remotely. In addition, it is a step toward health monitoring and requires patients to be part of it, which may help them learn to regulate recovery. Additionally, it is a friendly, easy-to-install and easy-to-use application compatible with both Android and IOS systems, so it can reach a population with fewer mobile phone capabilities.

### Clinical implications

ATOPE+ can be an excellent support tool for exercise programs in BCS, optimising physical exercise and improving adherence and safety. Additionally, it offers professionals a single, easy, remote and validated tool that assesses several parameters related to different systems and could identify risk profiles and target interventions to a particular problem. Lastly, it can be used together with other complementary tools, as it is not time-consuming and does not require patients to wear any device.

## Conclusion

ATOPE+ is a reliable and valid tool to monitor readiness in BCS, which could help rehabilitation professionals prescribe safer and optimal doses of exercise. This ensures that BCS have an adequate recovery period to induce compensation to meet the principles of training. As a new technology, it offers a more easy, efficient and inexpensive way of doing so. ATOPE+ is a realiable and valid tool to assess autonomic balance, sleep satisfaction, emotional distress in BCS. Therefore, it could be an excellent tool to support physical exercise programs in cancer survivor populations.

## Data Availability

The datasets used and/or analysed during the current study are available from the corresponding author on reasonable request.

## Abbreviations

ATOPE+: ATOPE+ mHealth system
BCS: Breast Cancer Survivors
CK: Creatine Kinase
ECG: Electrocardiogram
HRV: Heart Rate Variability
ICC: Intraclass correlation coefficient
Ln: Natural logarithm
LnRMSSD: Natural logarithm of the mean square root differences of the standard derivation
RMSSD: Mean square root differences of the standard derivation
VO2max: Maximal oxygen consumption
